# Supplementary material for: Increased burden of familial-associated early-onset cancer risk among minority Americans compared to non-Latino Whites
Source: eLife. 2021 Jun 22;10:e64793. doi: 10.7554/eLife.64793 (PMC8219377; doi:10.7554/eLife.64793)
Supplement: Supplementary file 1. — (A) Relative risks of the same type of early-onset cancer with the proband among siblings and mothers by ethnic group, 1989 to 2015, California, USA. (B) Relative risks of siblings and mothers for a specific type of early-onset cancer (diagnosed 0–26 years of age) given a proband with cancer, 1989–2015, California, USA. (C) Relative risks of any early-onset cancer (diagnosed at 0–26 years of age) for siblings and mothers of the same type of cancer with the proband given a proband with cancer by subgroups, 1989–2015, California, USA. (D) Relative risks of any early-onset cancer (diagnosed 0–26 years of age) among siblings and mothers by ethnic group, 1989–2015, California, USA. (E) Second primary malignancies in families exhibited familial risks and families did not exhibit familial risk. (F) Relative risks of second primary malignancies of the same type of early-onset cancer (diagnosed 0–26 years of age) with the first primary malignancy by ethnic groups, California, USA. [file elife-64793-supp1.docx]

**Supplemental File**

**Supplementary File 1a. Relative risks of the same type of early-onset cancer with the proband among siblings and mothers by ethnic group, 1989 to 2015, California, USA.**

|  | Overall | | | Non-Latino White | | | Latino all races | | |  |
| --- | --- | --- | --- | --- | --- | --- | --- | --- | --- | --- |
| Cancer of the proband ^†^ | No. of Probands | No. of affected relatives ^§^ | SIR (95% CI) | No. of Probands | No. of relatives ^§^ | SIR (95% CI) | No. of Probands | No. of affected relatives ^§^ | SIR (95% CI) | p-value ^‡^ |
| Hematologic cancers^¶^ | 11404 | 22 | 2.68*  (1.68, 4.06) | 2702 | 6 | 2.64  (0.97, 5.76) | 5484 | 7 | 1.56  (0.63, 3.21) | 0.505 |
| Solid cancers^¶^ | 17849 | 112 | 6.78*  (5.58, 8.16) | 5471 | 31 | 4.41*  (2.99, 6.25) | 7323 | 50 | 7.94*  (5.89, 10.47) | 0.012 |
| Leukemias | 8500 | 11 | 2.48*  (1.24, 4.45) | 1646 | ≦5 | 2.19  (0.27, 7.91) | 4374 | ≦5 | 1.45  (0.4, 3.72) | 0.996 |
| Lymphomas | 2928 | ≦5 | 5.9*  (1.61, 15.11) | 1060 | ≦5 | 9.04*  (1.86, 26.41) | 1121 | 0 |  | NA |
| CNS tumors | 5739 | 11 | 6.19*  (3.09, 11.08) | 1728 | ≦5 | 5.07*  (1.38, 12.98) | 2242 | ≦5 | 4.66  (0.96, 13.61) | 0.788 |
| Neuroblastoma | 1445 | ≦5 | 10.31  (0.26, 57.44) | 367 | 0 | NA | 490 | 0 |  | NA |
| Retinoblastoma | 718 | 10 | 454.55*  (217.97, 835.93) | 126 | ≦5 | 200*  (5.06, 1114.32) | 327 | 7 | 636.36*  (255.85, 1311.15) | 0.446 |
| Renal tumors | 1069 | ≦5 | 15.87  (0.4, 88.44) | 209 | 0 | NA | 442 | ≦5 | 38.46  (0.97, 214.29) | NA |
| Hepatic tumors | 414 | 0 | NA | 74 | 0 | NA | 200 | 0 |  | NA |
| Malignant bone tumors | 249 | 0 | NA | 73 | 0 | NA | 121 | 0 |  | NA |
| Sarcomas | 2915 | 10 | 31.45*  (15.08, 57.83) | 816 | ≦5 | 18.87*  (2.28, 68.16) | 1259 | ≦5 | 35.97*  (11.68, 83.94) | 0.687 |
| GCT | 2423 | ≦5 | 7.71*  (1.59, 22.54) | 672 | 0 | NA | 1270 | ≦5 | 8.62*  (1.04, 31.14) | NA |
| Epithelial neoplasms | 3050 | 24 | 40.68*  (26.06, 60.53) | 1458 | 9 | 21.33*  (9.75, 40.49) | 1046 | 9 | 55.56*  (25.4, 105.46) | 0.065 |
| Other | 255 | 0 | NA | 79 | 0 | NA | 90 | 0 | NA | NA |

^†^ Cancers were classified into subgroups as defined by the International Classification of Childhood Cancer, Third edition (ICCC-3, November 2012) (<https://seer.cancer.gov/iccc/iccc3.html>).

^§^ Affected relatives include mother and siblings of the proband diagnosed with early-onset cancer under 26 years of age.

^‡^ p-value comparing the SIRs between non-Latino White and Latino all races using an approximate Chi-squared test.

^¶^ Hematologic cancers include group 1, leukemias, myeloproliferative diseases, and myelodysplastic diseases; group 2, lymphomas and reticuloendothelial neoplasms. Solid cancers include I group 3, CNS and miscellaneous intracranial and intraspinal neoplasms; group 4, neuroblastoma and other peripheral nervous cell tumors; group 5, retinoblastoma; group 6, renal tumors; group 7, hepatic tumors; group 8, malignant bone tumors; group 9, soft tissue and other extraosseous sarcomas; group 10, germ cell tumors, trophoblastic tumors, and neoplasms of gonads; group 11, other malignant epithelial neoplasms and malignant melanomas; group 12, other and unspecified malignant neoplasms.

SIR, Standardized incidence ratio. CI, confidence interval.

* Statistically significant standardized incidence ratio with p<0.05 assuming a Poisson distribution.

**Supplemental File 1b. Relative risks of siblings and mothers for a specific type of early-onset cancer (diagnosed 0 to 26 years of age) given a proband with cancer, 1989 to 2015, California, USA.**

| **Cancer of the proband** ^†^ | **Cancer of the relative** ^§^ | **No. of affected relatives** ^§^ | **SIR (95% CI)** |
| --- | --- | --- | --- |
|  |  |  |  |
| **Leukemias** | Leukemias | 11 | 2.48* (1.24, 4.45) |
|  | Lymphomas | 6 | 2.46 (0.9, 5.35) |
|  | CNS tumors | 6 | 2.02 (0.74, 4.4) |
|  | Hepatic tumors | ≦5 | 0.41 (0.01, 2.28) |
|  | Sarcomas | 8 | 7.36* (3.18, 14.5) |
|  | GCT | 6 | 3.51* (1.29, 7.64) |
|  | Epithelial neoplasms | 13 | 5.37* (2.86, 9.18) |
| **Lymphomas** | Leukemias | 7 | 7.47* (3, 15.39) |
|  | Lymphomas | ≦5 | 5.9* (1.61, 15.11) |
|  | CNS tumors | ≦5 | 2.89 (0.35, 10.46) |
|  | Neuroblastoma | ≦5 | 15.38 (0.39, 85.72) |
|  | Retinoblastoma | ≦5 | 40* (1.01, 222.86) |
|  | Renal tumors | ≦5 | 12.99 (0.33, 72.36) |
|  | Sarcomas | ≦5 | 7.07 (0.86, 25.53) |
|  | GCT | ≦5 | 1.93 (0.05, 10.78) |
|  | Epithelial neoplasms | ≦5 | 2.67 (0.32, 9.65) |
| **CNS tumors** | Leukemias | ≦5 | 1.94 (0.63, 4.53) |
|  | Lymphomas | ≦5 | 1.31 (0.16, 4.72) |
|  | CNS tumors | 11 | 6.19* (3.09, 11.08) |
|  | Renal tumors | ≦5 | 4.29 (0.11, 23.91) |
|  | Sarcomas | 6 | 8.97* (3.29, 19.52) |
|  | GCT | ≦5 | 1.81 (0.22, 6.55) |
|  | Epithelial neoplasms | 10 | 6.37* (3.05, 11.71) |
|  | Other | ≦5 | 0.65 (0.02, 3.64) |
| **Neuroblastoma** | Lymphomas | ≦5 | 2.23 (0.06, 12.41) |
|  | Neuroblastoma | ≦5 | 10.31 (0.26, 57.44) |
|  | Sarcomas | ≦5 | 4.78 (0.12, 26.66) |
|  | GCT | ≦5 | 3.33 (0.08, 18.57) |
|  | Epithelial neoplasms | ≦5 | 5.09 (0.62, 18.38) |
|  | Other | ≦5 | 2.23 (0.06, 12.41) |
| **Retinoblastoma** | Lymphomas | ≦5 | 4.5 (0.11, 25.1) |
|  | Retinoblastoma | 10 | 454.55* (217.97, 835.93) |
|  | Malignant bone tumors | ≦5 | 4.5 (0.11, 25.1) |
|  | Sarcomas | ≦5 | 9.52 (0.24, 53.06) |
|  | Epithelial neoplasms | ≦5 | 5.24 (0.13, 29.17) |
| **Renal tumors** | Lymphomas | ≦5 | 3.02 (0.08, 16.83) |
|  | CNS tumors | ≦5 | 2.37 (0.06, 13.2) |
|  | Renal tumors | ≦5 | 15.87 (0.4, 88.44) |
|  | Sarcomas | ≦5 | 13.33* (1.61, 48.16) |
|  | GCT | ≦5 | 4.42 (0.11, 24.65) |
|  | Epithelial neoplasms | ≦5 | 9.17* (1.89, 26.81) |
| **Hepatic tumors** | Leukemias | ≦5 | 4.08 (0.1, 22.74) |
|  | Epithelial neoplasms | ≦5 | 9.52 (0.24, 53.06) |
| **Malignant bone tumors** | Leukemias | ≦5 | 14.49 (0.37, 80.75) |
|  | Epithelial neoplasms | ≦5 | 16.39 (0.41, 91.34) |
| **Sarcomas** | Leukemias | 8 | 7.01* (3.03, 13.82) |
|  | Lymphomas | ≦5 | 1.37 (0.03, 7.61) |
|  | CNS tumors | 7 | 8.62* (3.47, 17.76) |
|  | Neuroblastoma | ≦5 | 11.11 (0.28, 61.91) |
|  | Renal tumors | ≦5 | 19.8* (2.4, 71.53) |
|  | Sarcomas | 10 | 31.45* (15.08, 57.83) |
|  | GCT | ≦5 | 7.41* (2.02, 18.97) |
|  | Epithelial neoplasms | 6 | 7.56* (2.77, 16.45) |
|  | Other | ≦5 | 1.37 (0.03, 7.61) |
| **GCT** | Leukemias | ≦5 | 5.81* (1.58, 14.89) |
|  | Lymphomas | ≦5 | 4.06 (0.49, 14.65) |
|  | CNS tumors | ≦5 | 5.93* (1.22, 17.33) |
|  | Neuroblastoma | ≦5 | 19.23 (0.49, 107.15) |
|  | Renal tumors | ≦5 | 16.67 (0.42, 92.86) |
|  | Sarcomas | ≦5 | 9.66* (1.17, 34.9) |
|  | GCT | ≦5 | 7.71* (1.59, 22.54) |
|  | Epithelial neoplasms | ≦5 | 6.92* (1.89, 17.72) |
| **Epithelial neoplasms** | Leukemias | ≦5 | 8.39* (2.28, 21.47) |
|  | Lymphomas | ≦5 | 2.19 (0.06, 12.22) |
|  | CNS tumors | ≦5 | 7.56* (1.56, 22.08) |
|  | Neuroblastoma | ≦5 | 41.67* (1.05, 232.15) |
|  | Sarcomas | ≦5 | 10.99* (1.33, 39.7) |
|  | GCT | ≦5 | 2.63 (0.07, 14.66) |
|  | Epithelial neoplasms | 24 | 40.68* (26.06, 60.53) |
| **Other** | Lymphomas | ≦5 | 16.39 (0.41, 91.34) |
|  | CNS tumors | ≦5 | 14.29 (0.36, 79.59) |
|  | Neuroblastoma | ≦5 | 125* (3.16, 696.45) |
|  | Sarcomas | ≦5 | 37.04 (0.94, 206.36) |

^†^ Cancers were classified into subgroups as defined by the International Classification of Childhood Cancer, Third edition (ICCC-3, November 2012) (<https://seer.cancer.gov/iccc/iccc3.html>).

^§^ Affected relatives include mother and siblings of the proband diagnosed with early-onset cancer under 26 years of age.

SIR, Standardized incidence ratio. CI, confidence interval.

* Statistically significant standardized incidence ratio with p<0.05 assuming a Poisson distribution.

**Supplemental File 1c. Relative risks of any early-onset cancer (diagnosed at 0 to 26 years of age) for siblings and mothers of the same type of cancer with the proband given a proband with cancer by subgroups, 1989 to 2015, California, USA.**

|  |  | **All cancers** | | **The same type of cancer** | |
| --- | --- | --- | --- | --- | --- |
| **Cancer of the proband** ^†^ | **No. of probands** | **No. of affected relatives** ^§^ | **SIR**  **(95% CI)** | **No. of affected relatives** ^§^ | **SIR**  **(95% CI)** |
| **Lymphoid leukemias** | 6705 | 25 | 2.7* (1.91, 3.7) | ≦5 | 1.86 (0.6, 4.34) |
| **Acute myeloid leukemias** | 1317 | 9 | 4.61* (2.46, 7.89) | ≦5 | 27.27* (5.62, 79.7) |
| **Hodgkin lymphomas** | 1399 | 9 | 7.12* (3.79, 12.17) | ≦5 | 10.53* (1.27, 38.02) |
| **Non-Hodgkin lymphomas (except Burkitt lymphoma)** | 985 | ≦5 | 4.02* (1.48, 8.75) | ≦5 | 11.11 (0.28, 61.91) |
| **Ependymomas and choroid plexus tumor** | 561 | ≦5 | 2.46 (0.51, 7.19) | 0 | NA |
| **Astrocytomas** | 1965 | 11 | 3.6* (1.97, 6.03) | ≦5 | 7.69 (0.93, 27.79) |
| **Intracranial and intraspinal embryonal tumors** | 1010 | 6 | 2.67* (1.07, 5.5) | ≦5 | 11.11 (0.28, 61.91) |
| **Other gliomas** | 815 | 8 | 5.34* (2.31, 10.53) | ≦5 | 66.67* (8.07, 240.82) |
| **Other specified intracranial and intraspinal neoplasms** | 1470 | ≦5 | 5.48* (2.5, 10.4) | ≦5 | 22.22* (2.69, 80.27) |
| **Neuroblastoma and ganglioneuroblastoma** | 1409 | 6 | 2.06 (0.83, 4.25) | ≦5 | 9.09 (0.23, 50.65) |
| **Nephroblastoma and other nonepithelial renal tumors** | 1032 | 6 | 3.67* (1.68, 6.97) | ≦5 | 16.67 (0.42, 92.86) |
| **Rhabdomyosarcomas** | 719 | 11 | 8.46* (4.63, 14.2) | 0 | NA |
| **Fibrosarcomas to peripheral nerve sheath tumors to and other fibrous neoplasms** | 434 | 7 | 10.46* (4.21, 21.56) | ≦5 | 200* (24.22, 722.47) |
| **Other specified soft tissue sarcomas** | 1624 | 11 | 7.57* (4.56, 11.82) | ≦5 | 55.56* (18.04, 129.65) |
| **Malignant gonadal germ cell tumors** | 1030 | 12 | 5.44* (3.05, 8.97) | ≦5 | 6.67 (0.81, 24.08) |
| **Malignant melanomas** | 788 | ≦5 | 3.65 (0.75, 10.68) | ≦5 | 25 (0.63, 139.29) |
| **Other and unspecified carcinomas** | 1845 | 20 | 18.11* (12.13, 26.01) | 17 | 113.33* (66.02, 181.46) |

^†^Cancers were classified into subgroups as defined by the International Classification of Childhood Cancer, Third edition (ICCC-3, November 2012) (<https://seer.cancer.gov/iccc/iccc3.html>).

^§^ Affected relatives include mother and siblings of the proband diagnosed with early-onset cancer under 26 years of age.

SIR, Standardized incidence ratio. CI, confidence interval.

* Statistically significant standardized incidence ratio with p<0.05 assuming a Poisson distribution.

**Supplementary File 1d. Relative risks of any early-onset cancer (diagnosed 0 to 26 years of age) among siblings and mothers by ethnic group, 1989 to 2015, California, USA.**

|  | Non-Latino White | | | Latino all races | | | | Non-Latino API | | | | Non-Latino Black | | | |  |
| --- | --- | --- | --- | --- | --- | --- | --- | --- | --- | --- | --- | --- | --- | --- | --- | --- |
| Cancer of the proband ^†^ | No. of Probands | No. of affected relatives ^§^ | SIR  (95% CI) | No. of Probands | No. of affected relatives ^§^ | SIR (95% CI) | p-value ^‡^ | No. of Probands | No. of affected relatives ^§^ | SIR (95% CI) | p-value ^‡^ | No. of Probands | No. of affected relatives ^§^ | SIR (95% CI) | p-value ^‡^ |  |
| Overall | 8119 | 50 | 2.6*  (1.93, 3.43) | 12736 | 78 | 3.36* (  2.66, 4.19) | 0.183 | 1677 | 11 | 4.58*  (2.29, 8.2) | 0.128 | 1102 | 13 | 6.96*  (3.71, 11.91) | 0.002 |  |
| Hematologic cancers^¶^ | 2702 | 19 | 2.69*  (1.62, 4.2) | 5484 | 27 | 2.48*  (1.64, 3.61) | 0.910 | 679 | 8 | 7.56*  (3.26, 14.9) | 0.023 | 391 | ≦5 | 6.14*  (1.67, 15.73) | 0.242 |  |
| Solid cancers^¶^ | 5471 | 37 | 3.02*  (2.12, 4.16) | 7323 | 62 | 4.98*  (3.82, 6.39) | 0.019 | 1021 | 7 | 5.07*  (2.04, 10.44) | 0.306 | 719 | 9 | 7.35*  (3.36, 13.95) | 0.026 |  |
| Leukemias | 1646 | 11 | 2.11*  (1.05, 3.77) | 4374 | 21 | 2.35*  (1.45, 3.59) | 0.911 | 457 | 6 | 6.94*  (2.55, 15.12) | 0.032 | 205 | ≦5 | 6.58*  (1.36, 19.22) | 0.176 |  |
| Lymphomas | 1060 | 8 | 4.32*  (1.87, 8.52) | 1121 | 8 | 4.09*  (1.77, 8.07) | 0.887 | 226 | ≦5 | 20.3*  (5.53, 51.98) | 0.022 | 187 | ≦5 | 5.13  (0.13, 28.53) | 0.685 |  |
| CNS tumors | 1728 | 12 | 2.63*  (1.36, 4.59) | 2242 | 12 | 2.86*  (1.48, 4.99) | 0.999 | 311 | ≦5 | 7.14*  (1.47, 20.87) | 0.250 | 239 | ≦5 | 7.56*  (1.56, 22.08) | 0.216 |  |
| Neuroblastoma | 367 | ≦5 | 0.7  (0.02, 3.92) | 490 | ≦5 | 1.7  (0.21, 6.13) | 0.871 | 51 | ≦5 | 6.54  (0.17, 36.37) | 0.466 | 48 | 0 | NA | NA |  |
| Retinoblastoma | 126 | ≦5 | 3.65  (0.44, 13.18) | 327 | 9 | 12.31*  (5.63, 23.37) | 0.178 | 35 | 0 | NA | NA | 30 | ≦5 | 11.49  (0.29, 63.95) | 0.881 |  |
| Renal tumors | 209 | ≦5 | 2.64  (0.32, 9.54) | 442 | ≦5 | 2.91  (0.6, 8.5) | 0.728 | 28 | 0 | NA | NA | 53 | ≦5 | 8.85  (0.22, 49.24) | 0.850 |  |
| Hepatic tumors | 74 | 0 | NA | 200 | ≦5 | 2.43  (0.06, 13.52) | NA | 13 | 0 | NA | NA | 6 | 0 | NA | NA |  |
| Bone tumors | 73 | 0 | NA | 121 | ≦5 | 6.06  (0.15, 33.77) | NA | 13 | 0 | NA | NA | 17 | 0 | NA | NA |  |
| Sarcomas | 816 | 7 | 3.77*  (1.51, 7.76) | 1259 | 20 | 9.05*  (5.53, 13.98) | 0.062 | 160 | ≦5 | 4.46  (0.11, 24.84) | 0.681 | 132 | ≦5 | 10.68*  (2.2, 31.19) | 0.267 |  |
| GCT | 672 | ≦5 | 3.42  (0.93, 8.77) | 1270 | 10 | 6.2*  (2.97, 11.41) | 0.454 | 184 | ≦5 | 4.85  (0.12, 27.01) | 0.755 | 70 | 0 | NA | NA |  |
| Epithelial neoplasms | 1458 | 14 | 8.6*  (4.7, 14.43) | 1046 | 15 | 15.15*  (8.48, 24.99) | 0.176 | 239 | ≦5 | 13.25*  (1.6, 47.82) | 0.899 | 122 | ≦5 | 22.47*  (2.72, 81.13) | 0.450 |  |
| Other | 79 | ≦5 | 10.47*  (1.27, 37.83) | 90 | ≦5 | 5.85  (0.15, 32.58) | 0.924 | 8 | 0 | NA | NA | 19 | 0 | NA | NA |  |

^†^ Cancers were classified into subgroups as defined by the International Classification of Childhood Cancer, Third edition (ICCC-3, November 2012) (<https://seer.cancer.gov/iccc/iccc3.html>).

^§^ Affected relatives include mother and siblings of the proband diagnosed with early-onset cancer under 26 years of age.

^‡^ p-value comparing the SIRs between non-Latino White and Latino all races using an approximate Chi-squared test.

^¶^ Hematologic cancers include group 1, leukemias, myeloproliferative diseases, and myelodysplastic diseases; group 2, lymphomas and reticuloendothelial neoplasms. Solid cancers include I group 3, CNS and miscellaneous intracranial and intraspinal neoplasms; group 4, neuroblastoma and other peripheral nervous cell tumors; group 5, retinoblastoma; group 6, renal tumors; group 7, hepatic tumors; group 8, malignant bone tumors; group 9, soft tissue and other extraosseous sarcomas; group 10, germ cell tumors, trophoblastic tumors, and neoplasms of gonads; group 11, other malignant epithelial neoplasms and malignant melanomas; group 12, other and unspecified malignant neoplasms.

SIR, Standardized incidence ratio. CI, confidence interval.

* Statistically significant standardized incidence ratio with p<0.05 assuming a Poisson distribution.

**Supplemental File 1e. Second primary malignancies in families exhibited familial risks and families did not exhibit familial risk.**

|  | **Family exhibited familial risks** | **Family did not exhibit familial risk** |
| --- | --- | --- |
| **No. of SPMs** | 14 | 373 |
| **Total No. of family members** | 2,432 | 119,136 |
| **No. of SPMs per family member** ^†^ | 0.58% | 0.31% |
| **Average family size** | 4.35 people/family | 4.17 people/family |
| **Average time between FPM and SPM diagnosis** | 6.69 years | 6.35 years |
| **Average time from FPM diagnosis to the end of study (2015)** | 12.23 years | 11.74 years |

FPM, first primary malignancy. SPM, second primary malignancy.

^†^ No. of SPMs per family member was compared with a Chi-squared test. Families that exhibited familial risks have higher proportion of SPMs compared to families that did not exhibit familial risk (Chi^2^= 5.13; p= 0.023).

**Supplemental File 1f. Relative risks of second primary malignancies of the same type of early-onset cancer (diagnosed 0 to 26 years of age) with the first primary malignancy by ethnic groups, California, USA.**

|  | **Overall** | | | **Non-Latino White** | | | **Latino (all races)** | | |  |
| --- | --- | --- | --- | --- | --- | --- | --- | --- | --- | --- |
| **Cancer of the proband** ^†^ | **No. of FPMs** | **No. of SPMs** | **SIR**  **(95% CI)** | **No. of FPMs** | **No. of SPMs** | **SIR**  **(95% CI)** | **No. of FPMs** | **No. of SPMs** | **SIR**  **(95% CI)** | **p-value ^‡^** |
| **Hematologic cancers^¶^** | 11,553 | 50 | 6.1*  (4.53, 8.04) | 2,727 | 15 | 6.61*  (3.7, 10.9) | 5,528 | 19 | 4.23*  (2.55, 6.61) | 0.262 |
| **Solid cancers^¶^** | 18,893 | 30 | 1.82*  (1.22, 2.59) | 5,673 | 13 | 1.85  (0.98, 3.16) | 7,551 | 13 | 2.06*  (1.1, 3.53) | 0.938 |
| **Leukemias** | 8,543 | 41 | 9.26*  (6.65, 12.56) | 1,649 | 11 | 12.05*  (6.01, 21.56) | 4,385 | 16 | 5.82*  (3.32, 9.44) | 0.094 |
| **Lymphomas** | 2,960 | 9 | 13.27*  (6.07, 25.2) | 1,063 | ≦5 | 12.05*  (3.28, 30.85) | 1,124 | ≦5 | NA | 0.768 |
| **CNS tumors** | 5,877 | 29 | 16.32*  (10.93, 23.44) | 1,765 | 12 | 15.21*  (7.86, 26.57) | 2,277 | 11 | 17.08*  (8.53, 30.56) | 0.943 |
| **Neuroblastoma** | 1,450 | ≦5 | 20.62*  (2.5, 74.48) | 369 | ≦5 | NA | 491 | 0 | NA | NA |
| **Retinoblastoma** | 728 | 0 | NA | 127 | 0 | NA | 334 | 0 | NA | NA |
| **Renal tumors** | 1,078 | 0 | NA | 209 | 0 | NA | 444 | 0 | NA | NA |
| **Hepatic tumors** | 417 | 0 | NA | 74 | 0 | NA | 200 | 0 | NA | NA |
| **Malignant bone tumors** | 250 | 0 | NA | 73 | 0 | NA | 121 | 0 | NA | NA |
| **Sarcomas** | 3,028 | 16 | 50.31*  (28.76, 81.71) | 850 | ≦5 | 47.17*  (15.32, 110.08) | 1,297 | 7 | 50.36*  (20.25, 103.76) | 0.858 |
| **GCT** | 2,468 | ≦5 | 5.14  (0.62, 18.57) | 679 | 0 | NA | 1,287 | ≦5 | 8.62*  (1.04, 31.14) | NA |
| **Epithelial neoplasms** | 3,479 | 10 | 16.95*  (8.13, 31.17) | 1,489 | 6 | 14.22*  (5.22, 30.95) | 1,057 | ≦5 | 24.69*  (6.73, 63.22) | 0.605 |
| **Other** | 261 | 0 | NA | 79 | 0 | NA | 90 | 0 | NA | NA |

SIR, Standardized incidence ratio. CI, confidence interval. FPM, first primary malignancy. SPM, second primary malignancy.

^†^ Cancers were classified into subgroups as defined by the International Classification of Childhood Cancer, Third edition (ICCC-3, November 2012) (<https://seer.cancer.gov/iccc/iccc3.html>).

^‡^ p-value comparing the SIRs between non-Latino White and Latino all races using an approximate Chi-squared test.

^¶^ Hematologic cancers include group 1, leukemias, myeloproliferative diseases, and myelodysplastic diseases; group 2, lymphomas and reticuloendothelial neoplasms. Solid cancers include I group 3, CNS and miscellaneous intracranial and intraspinal neoplasms; group 4, neuroblastoma and other peripheral nervous cell tumors; group 5, retinoblastoma; group 6, renal tumors; group 7, hepatic tumors; group 8, malignant bone tumors; group 9, soft tissue and other extraosseous sarcomas; group 10, germ cell tumors, trophoblastic tumors, and neoplasms of gonads; group 11, other malignant epithelial neoplasms and malignant melanomas; group 12, other and unspecified malignant neoplasms.

SIR, Standardized incidence ratio. CI, confidence interval. FPM, first primary malignancy. SPM, second primary malignancy.

* Statistically significant standardized incidence ratio with p<0.05 assuming a Poisson distribution.
