# Supplementary material for: Increased burden of familial-associated early-onset cancer risk among minority Americans compared to non-Latino Whites
Source: eLife. 2021 Jun 22;10:e64793. doi: 10.7554/eLife.64793 (PMC8219377; doi:10.7554/eLife.64793)
Supplement: Transparent reporting form [file elife-64793-transrepform.docx]

***eLife’s* transparent reporting form**

We encourage authors to provide detailed information *within their submission* to facilitate the interpretation and replication of experiments. Authors can upload supporting documentation to indicate the use of appropriate reporting guidelines for health-related research (see [EQUATOR Network](http://www.equator-network.org/%20)), life science research (see the [BioSharing Information Resource](https://biosharing.org/)), or the [ARRIVE guidelines](http://www.plosbiology.org/article/info:doi/10.1371/journal.pbio.1000412) for reporting work involving animal research. Where applicable, authors should refer to any relevant reporting standards documents in this form.

If you have any questions, please consult our Journal Policies and/or contact us: [editorial@elifesciences.org](mailto:editorial@elifesciences.org).

**Sample-size estimation**

- You should state whether an appropriate sample size was computed when the study was being designed
- You should state the statistical method of sample size computation and any required assumptions
- If no explicit power analysis was used, you should describe how you decided what sample (replicate) size (number) to use

Please outline where this information can be found within the submission (e.g., sections or figure legends), or explain why this information doesn’t apply to your submission:

No explicit power analysis was used. We expected significant results because the previous Sweden (Sud et. al., 2019) and Utah studies (Curtin et. al., 2013) found significantly increased familial risk for certain cancers given a smaller population but longer time of follow-up. The population is around 10 million in Sweden, 3 million in Utah and 40 million in California. Although the Swedish study had a longer time of follow-up since 1958 compared to the 1989 in our study, we still expect to observe some significant results given the large population in California, and in addition the power to detect differences between the two largest racial/ethnic birth populations in California – Latinos, and non-Latino whites.

**Replicates**

- You should report how often each experiment was performed
- You should include a definition of biological versus technical replication
- The data obtained should be provided and sufficient information should be provided to indicate the number of independent biological and/or technical replicates
- If you encountered any outliers, you should describe how these were handled
- Criteria for exclusion/inclusion of data should be clearly stated
- High-throughput sequence data should be uploaded before submission, with a private link for reviewers provided (these are available from both GEO and ArrayExpress)

Please outline where this information can be found within the submission (e.g., sections or figure legends), or explain why this information doesn’t apply to your submission:

Not applicable. Our paper is an observational study connecting existing data from several population-based registry systems, calculating rates of cancer incidence among families. We did not perform experiments.

**Statistical reporting**

- Statistical analysis methods should be described and justified
- Raw data should be presented in figures whenever informative to do so (typically when N per group is less than 10)
- For each experiment, you should identify the statistical tests used, exact values of N, definitions of center, methods of multiple test correction, and dispersion and precision measures (e.g., mean, median, SD, SEM, confidence intervals; and, for the major substantive results, a measure of effect size (e.g., Pearson's r, Cohen's d)
- Report exact p-values wherever possible alongside the summary statistics and 95% confidence intervals. These should be reported for all key questions and not only when the p-value is less than 0.05.

Please outline where this information can be found within the submission (e.g., sections or figure legends), or explain why this information doesn’t apply to your submission:

- The statistical methods are described and justified in the methods section.
- We are prohibited by the State of California in sharing raw data that belongs to the State. This data can be accessed by other researchers from the same sources as we describe in the paper by qualified researchers who apply to the State IRB.
- The details of statistical tests along with the R codes are included in the additional data file.
- Exact p-values were calculated, and very small p-values less than 0.001 are denoted as “<0.001”.

(For large datasets, or papers with a very large number of statistical tests, you may upload a single table file with tests, Ns, etc., with reference to sections in the manuscript.)

**Group allocation**

- Indicate how samples were allocated into experimental groups (in the case of clinical studies, please specify allocation to treatment method); if randomization was used, please also state if restricted randomization was applied
- Indicate if masking was used during group allocation, data collection and/or data analysis

Please outline where this information can be found within the submission (e.g., sections or figure legends), or explain why this information doesn’t apply to your submission:

We grouped the subjects by self-reported race/ethnicity to identify the variation in familial risk by race/ethnicity group.

Masking is not applicable.

**Additional data files (“source data”)**

- We encourage you to upload relevant additional data files, such as numerical data that are represented as a graph in a figure, or as a summary table
- Where provided, these should be in the most useful format, and they can be uploaded as “Source data” files linked to a main figure or table
- Include model definition files including the full list of parameters used
- Include code used for data analysis (e.g., R, MatLab)
- Avoid stating that data files are “available upon request”

Please indicate the figures or tables for which source data files have been provided:

We apologize for not being able to upload complete data files because the State of California forbids researchers from publicizing information of cancer patients. When publishing aggregate data, any cell with less than 5 subjects should not be included. Instead, we have uploaded a blinded version of datasets used to generate the figures in this study to Dryad.
